# Fibroblast-Derived Small Extracellular Vesicles Promote M2 Macrophage Polarization and PD-L1 Upregulation in Mycosis Fungoides

**DOI:** 10.3390/cancers18132140

**Published:** 2026-07-02

**Authors:** Haneen Khoury, Emmilia Hodak, Jamal Knaneh, Batia Gorovitz-Harris, Feba John, Coral Arkin, Maya Bal, Anna Aronovich, Aladin Samara, Iris Amitay-Laish, Hadas Prag-Naveh, Lilach Moyal

**Affiliations:** 1 Felsenstein Medical Research Center, Rabin Medical Center, Petah Tikva 4941492, Israel; haneen.kh.97.hk@gmail.com (H.K.); hodakemmilia@gmail.com (E.H.); jamal.knaneh@gmail.com (J.K.); gorovitz@tauex.tau.ac.il (B.G.-H.); febajohn9595@gmail.com (F.J.); coralarkin1@gmail.com (C.A.); balmaya8@gmail.com (M.B.); anaronovich@gmail.com (A.A.); aladin.samara@gmail.com (A.S.); 2 Gray Faculty of Medicinal and Health Sciences, Tel Aviv University, Tel Aviv 6997801, Israel; amitaylaishiris@gmail.com (I.A.-L.); hadas.prag.naveh@gmail.com (H.P.-N.); 3 Davidoff Cancer Center, Rabin Medical Center, Petah Tikva 4941492, Israel; 4 Faculty of Life Sciences, Tel Aviv University, Tel Aviv 6997801, Israel; 5 Division of Dermatology, Rabin Medical Center, Beilinson Hospital, Petah Tikva 4941492, Israel; 6 Institute of Hematology, Davidoff Cancer Center, Rabin Medical Center, Petah Tikva 4941492, Israel

**Keywords:** exosomes, small extracellular vesicles, cutaneous T cell lymphoma, mycosis fungoides, cancer-associated fibroblasts

## Abstract

**Introduction**: Cutaneous T cell lymphoma (CTCL), most commonly known as mycosis fungoides (MF), is characterized by an increasingly immunosuppressive tumor microenvironment (TME) as the disease progresses. Cancer-associated fibroblasts (CAFs) are key stromal components that support a permissive niche, in part through the secretion of small extracellular vesicles (sEVs), predominantly exosomes, that mediate intercellular communication. We investigated the immunomodulatory role of exosome-enriched sEVs derived from MF fibroblasts (MF-Fs) compared to normal fibroblasts (N-Fs). **Materials and Methods**: Primary MF-Fs from early-stage MF biopsies and N-Fs from healthy skin were cultured in vitro. sEVs enriched with exosomes were isolated by ultracentrifugation and characterized by flow cytometry (CD81), electron microscopy, Nanosight analysis, and protein quantification, and their uptake by normal peripheral blood mononuclear cells (nPBMCs) was confirmed using PKH26-labeled sEVs. nPBMCs, monocytes, CD4^+^ and CD8^+^ T cells from healthy donors were exposed to MF-F or N-F sEVs. Cell viability was assessed using MTT and trypan blue exclusion assays. Mass cytometry (CyTOF) profiled immune subsets and regulatory proteins for preliminary observation. Monocyte polarization was evaluated by flow cytometry for M1 (CD80, CD86) and M2 (CD163, CD206) markers and PD-L1 expression; M1/M2-associated cytokines and sEV-microRNAs were quantified by qRT-PCR. **Results**: Both MF-F and N-F sEVs were internalized by nPBMCs and reduced their viability, with a more pronounced effect observed for MF-F sEVs. In nPBMCs, MF-F sEVs also increased the frequency of M2-like macrophages, decreased M1 polarization, and enhanced PD-L1 expression. In primary monocytes, MF-F- compared with N-F-derived sEVs upregulated M2-associated cytokines (IL-10, TGF-β), increased PD-L1 expression, and generated M2-like cells that suppressed CD4^+^ and CD8^+^ T cell viability. **Conclusions**: MF-F sEVs promote an immunosuppressive TME and represent potential therapeutic or biomarker targets in MF.

## Simple Summary

Mycosis fungoides (MF) is a type of skin lymphoma that develops in a complex environment made up of cancer cells and supporting cells in the skin. Among these supporting cells are fibroblasts, which can be “educated” by the tumor and then help it grow and evade the immune system. Fibroblasts release tiny particles called sEVs (enriched with exosomes), which carry signals to immune cells, but their role in MF has not yet been studied. In this study, we cultured fibroblasts from lesional biopsies of patients with early-stage MF and from healthy donors. We isolated their sEVs enriched with exosomes and tested how they affected immune cells from healthy donors. The sEVs from MF fibroblasts skewed immune cells called monocytes toward a suppressive state, with higher levels of molecules (IL-10, TGF-β and PD-L1) that turn down immune responses. These conditioned cells then reduced the survival of both CD4^+^ and CD8^+^ T cells, which are important for fighting cancer. Our results suggest that fibroblast-derived sEVs help create an immunosuppressive environment in MF and may contribute to the disease’s ability to escape immune control. Targeting these sEVs could offer new ways to diagnose or treat this skin lymphoma.

## 1. Introduction

Cutaneous T cell lymphoma (CTCL) is a non-Hodgkin lymphoma characterized by malignant T-cell infiltration in the skin [1]. Mycosis fungoides (MF), the most common type of CTCL, and Sézary Syndrome (SS), the leukemic variant, are characterized by tumor cells typically displaying a CD3^+^CD4^+^CD8^−^ phenotype. The tumor microenvironment (TME) plays a crucial role in disease evolution, undergoing a shift from a Th1 to Th2 cytokine profile during MF progression [2]. A major component of the TME are cancer-associated fibroblasts (CAFs), activated fibroblasts that promote tumor progression by: enhancing proliferation and therapy resistance and by creating a favorable niche through immune regulation, angiogenesis, and extracellular matrix (ECM) remodeling [3]. CAFs originate from various cell types, including normal fibroblasts, adipocytes, pericytes, and mesenchymal stem cells [4]. CAFs regulate the TME by secreting growth factors, cytokines, chemokines, small extracellular vesicles (sEVs), including exosomes, and extracellular matrix (ECM) components, thereby influencing tumor progression, immune evasion, and therapy resistance [3]. CAFs interact with immune cells, including T cells, NK cells, macrophages, and neutrophils, to promote an immunosuppressive TME [5,6]; they induce M2 macrophage and mesenchymal stem cell-derived immunosuppressive phenotypes [7], recruit Tregs and Th17 cells, and nodulate NK-cell function, ultimately supporting tumor growth and metastasis [8].

We were the first to characterize CAFs in MF [9], demonstrating increased expression of FAP-α, CXCL12, collagen XI, and MMP2, a higher proliferation rate than normal fibroblasts, and enhanced MF cell line migration and chemo-resistance via the CXCL12/CXCR4 axis. A recent study showed that tumor cells and CAFs establish a mutual positive feedback loop in MF, whereby malignant T cells induce CAF differentiation, and CAFs, in turn, enhance the invasiveness and metastatic potential of these T cells through IL-6/JAK2/STAT3/SOX4 or IL-6/HIF-1a/SOX4 signaling pathways [10].

One of the main mechanisms by which CAFs shape the TME is through sEVs, particularly exosomes [11], which are small EVs (30–150 nm) secreted by most cells and present in various bodily fluids. They are formed by inward budding of the endosomal membrane, leading to multivesicular body (MVB) formation and subsequent release of intraluminal vesicles (ILVs) as exosomes. sEVs and mainly exosomes carry bioactive molecules, including RNA, proteins, and lipids, with distinct cargo which internalizes into the cytosol of recipient cells. Tumor exosomes (TEX) [12] can be both immunosuppressive and immunostimulatory, influencing tumor immunity by modulating antigen presentation and immune cell function. They promote tumor growth by enhancing proliferation, enabling evasion of apoptosis, and reprogramming cells locally and at distant sites. TEX suppress immune responses by inhibiting T cells, inducing Treg differentiation, blocking NK-cell cytotoxicity, and promoting immune escape.

CAF-derived exosome-enriched sEVs [13,14] shape an immunosuppressive TME by delivering molecules, including miRNAs and immune checkpoint proteins, that suppress CD8^+^ T cell proliferation and infiltration of NK-cell function; promote myeloid-derived suppressor cells (MDSCs), T cell exhaustion, M2 macrophage polarization, and Treg conversion; and alter antigen presentation thereby reducing immune priming efficiency. We were the first to characterize exosomes/sEVs in MF, identifying a distinctive miRNA signature with high levels of miR-155 and miR-1246 [15]. Exosome/sEV miR-155, as well as plasma exosomes from MF patients, promoted migration of recipient cells. Furthermore, the levels of miR-155 and miR-1246 in plasma of MF patients correlated with skin tumor burden. To date, there are no published data on sEVs derived from MF-associated CAFs and their role in regulating the immune TME.

We aim to investigate the immune-regulating role of exosome-enriched sEVs derived from primary fibroblasts of MF compared to normal fibroblasts.

## 2. Materials and Methods

**Ethics statement:** The study was conducted in accordance with the declaration of Helsinki and was approved by the Rabin Medical Center Helsinki institutional committee (0264-17-RMC, 29 October 2017). All procedures involving human participants complied with the applicable guidelines and regulations of the Rabin Medical Center Helsinki board. Residual blood samples were obtained from routine blood donations and were used for research purposes only after donors had provided written informed consent specifically allowing secondary use of leftover samples, in accordance with national regulations and approval of the Rabin Medical Center Helsinki institutional committee (6515-11-RMC, 9 October 2011).

**Isolation of normal mononuclear cells from peripheral blood (nPBMCs)**: Buffy coats from leftover healthy donor blood donations were diluted 1:1 with sterile PBS (Biological Industries, Beit HaEmek, Israel) at room temperature (RT). An equal volume of Ficoll (Axis Shield, Dundee, UK) was carefully layered using a Pasteur pipette, followed by centrifugation at 1600 rpm for 30 min at RT with the brake off. The lymphocyte layer (white ring) was collected while avoiding red blood cells and granulocytes, then washed twice with PBS (1300 rpm, 5 min, RT), discarding the upper layer each time. The cells were resuspended in 8 mL RPMI (Biological Industries), centrifuged (1300 rpm, 5 min, RT), and adjusted to a known volume for counting. Lymphocytes were cultured at 1 × 10^6^ cells/mL in RPMI 1640 supplemented with 1% L-glutamine (Biological Industries), 1% penicillin/streptomycin (Biological Industries), and 10% human serum (Sigma-Aldrich, St. Louis, MO, USA).

**Isolation of monocyte cells**: Monocytes (CD14^+^ CD16^−^) were isolated from fresh nPBMCs by immunomagnetic negative selection using the EasySep Human Monocyte Isolation Kit (STEMCELL TECHNOLOGIES, Vancouver, BC, Canada). The cells were cultured in RPMI. The purity of the isolation was verified by FACS before and after enrichment with the use of R718 CD16 (BD Pharmingen, San Diego, CA, USA) and BV510 CD14 (BD Pharmingen).

**Isolation of T cells**: CD4^+^ T cells and CD8^+^ T cells were isolated from buffy coats of healthy donors by negative selection using the Rosettesep Human CD4^+^ T cell Enrichment Cocktail (STEMCELL TECHNOLOGIES) and the Rosettesep human CD8^+^ T cell Enrichment Cocktail (STEMCELL TECHNOLOGIES). The cells were cultured in complete RPMI medium with IL-2 (50 IU/Ml). The isolations were verified by FACS of cell samples before and after enrichment with FITC anti-human CD4 (Biotest, Dreieich, Germany), and APC-Vio 770 anti-human CD8 (MiltenyiBiotec’s, Bergisch Gladbach, Germany) antibodies.

**Establishment of primary fibroblasts from human skin samples**: Punch biopsies (4 mm) were taken from ~12 lesions of early-stage MF patients at the time of diagnosis, and ~12 healthy subjects undergoing post-bariatric reduction abdominoplasty. MF tissues and normal skin were minced and digested with a mixture of 2% collagenase II (Thermo Fisher Scientific, Waltham, MA, USA) and deoxyribonuclease (Worthington-Biochem, Lakewood, NJ, USA). Primary cultures of fibroblasts in passages 2–6 were used for the experiments. The cells were cultured in DMEM.

**Isolation of exosome-enriched sEVs from primary fibroblast culture**: N-F and MF-F cells were cultured to 80% cell confluence in medium supplemented with sEV-free FBS for two days. Fibroblast conditioned media, ~400 mL, was collected and subjected to sequential centrifugation: 1000× *g* for 10 min at 4 °C to remove dead cells, followed by 2000× *g* for 10 min at 4 °C to eliminate large debris. The supernatant was then centrifuged at 18,000× RPM for 30 min at 4 °C and filtered through a 0.22 μm Steriflip-GP filter to remove remaining debris and large vesicles. The filtered media was ultracentrifuged at 110,000× *g* for 90 min at 4 °C in a SW 40 Ti rotor using an Optima XPN 90 ultracentrifuge (Beckman Coulter, Brea, CA, USA). The sEV pellet was resuspended in PBS, centrifuged again at 110,000× *g* for 90 min, and the supernatant was discarded. The exosome pellet was resuspended in ~200 µL of PBS and either used immediately or stored at −80 °C. sEVs derived from MF-Fs and N-Fs showed similar particle concentrations by Nanosight analysis of ~10^11^ particles/mL (Malvern Panalytical, Malvern, UK).

**FACS analysis of CD81 on sEVs**: A total of 10 μL of latex FACS beads were incubated for 15 min at RT with 40 μL of sEVs (~4 × 10^9^ particles) derived from N-Fs and MF-Fs. The mixture was diluted with PBS and rotated overnight at 4 °C. sEV–bead complexes were centrifuged at 4500× *g* for 5 min at 4 °C, and the supernatant was discarded. To block non-specific binding, 200 μL of 100 mM glycine (Bio-Lab, Jerusalem, Israel) was added, vortexed, incubated for 15 min at RT, and centrifuged again. The pellet was resuspended in PBS containing APC-CD81 (Abcam, Cambridge, UK) or APC IgG-1 isotype (BD) and incubated for 12 min at 4 °C. After washing with PBS and centrifugation, the beads were resuspended in 350 μL of sample buffer and analyzed by FACS (red laser), gating 10,000 events.

**Transmission electron microscopy (TEM)**: sEVs were adsorbed onto formvar/carbon-coated nickel grids, which were pre-treated with UV light for 15 min. The grids were incubated with 15 µL of the sEV sample (~1.5 × 10^9^ particles) for 10 min, fixed with 2.5% glutaraldehyde for 10 min, and stained with 2% uranyl acetate for 10 min. Samples were visualized using a JEM 1400 Plus transmission electron microscope (Jeol, Akishima, Japan), and images were captured with SIS Mega View III and iTEM 5.2 imaging software (Olympus, Tokyo, Japan).

**Nanoparticle tracking analysis (NTA)**: sEVs were diluted 1:1000 in PBS and analyzed using a NanoSight NS300 instrument following the manufacturer’s instructions. The NTA 3.2 software tracked individual vesicles via Brownian motion, across 5 recordings, recorded them in real-time videos of 1498 frames, and calculated exosome size and concentration.

**Lysis of sEVs for protein extraction and quantification**: sEVs suspended in PBS were lysed by mixing the sEV suspension 1:1 with RIPA buffer (Sigma-Aldrich) supplemented with complete™ Mini, EDTA-free Protease Inhibitor Cocktail (Roche Diagnostics, Basel, Switzerland). The mixture was incubated at RT for 5 min and then sonicated on ice for 30 s.

The protein concentration of the sEV lysates was determined using the Pierce™ BCA Protein Assay Kit (Thermo Fisher Scientific, Cat. No. 23227) according to the microplate protocol. Absorbance was measured at 562 nm using a Synergy H1 microplate reader (BioTek Instruments, Winooski, VT, USA) operated with Gen5 software (version 3.11). Protein concentrations were calculated by interpolation from a BSA standard curve after subtraction of the blank.

**Western blot**: MF-F and N-F sEVs, along with their corresponding parental cells, were lysed in RIPA buffer. A total of 20 µg of protein per sample was separated by SDS-PAGE, followed by dry transfer. The membrane was probed with an anti-calnexin antibody (Abcam-ab 75801).

**Proteomic mass spectrometry of MF-F and N-F sEVs**: A total of 200 µL of sEVs (~2 × 10^10^ particles) isolated from 4 MF-F and 3 N-F cells were analyzed by mass spectrometry at the Smoler Proteomics Center in the core facility of the Technion. sEV samples were boiled, sonicated, precipitated and then digested. The peptides were resolved by reverse-phase chromatography on 0.075 × 200 mm fused silica capillaries (J&W Scientific, Folsom, CA, USA) packed with Reprosil reversed phase material (Dr Maisch GmbH, Ammerbuch-Entringen, Germany). The peptides were eluted and mass spectrometry was performed by a Q-Exactive plus mass spectrometer (QE, Thermo) in a positive mode using repetitively full MS scan followed by high-energy collision dissociation (HCD) of the 10 most dominant ions selected from the first MS scan. The mass spectrometry data was analyzed using the Protein Discoverer 1.4 software with two search algorithms: Sequest (Thermo) and Mascot (Matrix science, London, UK) were used to search against the human proteome from the Uniprot database with a mass tolerance of 20 ppm for the precursor masses and 20 ppm for the fragment ions. Peptide- and protein-level false discovery rates (FDRs) were filtered to 1% using the target-decoy strategy. The protein table was filtered to eliminate identifications from the reverse database, common contaminants and single peptide identifications. The data was semi-quantified based on extracted ion currents (XICs) of peptides. The area of the protein is the average of the three most intense peptides from each protein.

**Tracking exosome internalization**: sEVs were labeled with PKH26 (Sigma-Aldrich). A total of 50 µL of sEVs was mixed with 1500 µL of Diluent C and 4 µL of PKH26 dye, then incubated for 10 min at 4 °C. Labeled sEVs were washed twice by ultracentrifugation at 100,000× *g* with 60 mL PBS. PBS wash from the washed sEVs was used as a negative control to exclude free dye contamination. A total of 0.5 mL of nPBMCs 5 × 10^5^ cells/mL in RPMI were incubated with 15 µL of PKH26-labeled sEVs (~1.5 × 10^9^ particles) and with 15 µL of the wash supernatant for 24 h. nPBMCs were centrifuged using Cytospin, fixed with 4% PFA on ice for 10 min, and washed three times with PBS. DAPI mounting (Sigma-Aldrich) was applied for 20 min. Images were taken with an AXIOIMAGER Z2 microscope (Carl Zeiss Microscopy GmbH, Jena, Germany) equipped with AXIOCAM 506 MONO and AXIOCAM 503 cameras under ×40 magnification with a red laser.

**MTT cell viability assay**: A total of 100 µL of 4 × 10^6^ cells/mL of nPBMCs in RPMI were seeded in 96-well plates with or without 20 µL of sEVs (~2 × 10^9^ particles) for 72 h. Then, 10 µL of MTT (5 mg/mL) (Sigma-Aldrich) was added to each well and incubated for 4 h. Afterward, 140 µL of 0.1 N HCl in isopropanol (Sigma-Aldrich) was added to solubilize the formazan crystals. Cell viability was measured using an ELISA reader at 570 nm with background subtraction at 630–690 nm.

**Mass cytometry by time of flight (cyTOF)**: Then, 3 × 10^6^ nPBMCs from each donor were incubated with 200 µL of sEVs (~2 × 10^10^ particles) for 24 h. The cells were washed with CSM and incubated for 15 min in 500 μL of 250 nM rhodium solution (Fluidigm Corporation, South San Francisco, CA, USA) at RT, for viability staining. A mix of isotope-labeled antibodies were used for staining surface proteins in a total reaction volume of 100 μL; the cells were incubated at RT for 30 min, washed and permeabilized with a BD TF kit for 45 min at 4 °C. After permeabilization, the cells were incubated with isotope-labeled antibodies to intracellular proteins for 45 min on ice. The cells were then fixed in 1.6% PFA for 10 min, washed and resuspended in 125 nM Ir DNA intercalator (Fluidigm Corporation), and incubated overnight at 4 °C. Prior to CyTOF acquisition, samples were washed once in CSM, and twice more in Cell Acquisition Solution (Fluidigm Corporation). Next, they were passed through a cell strainer and each sample was spiked with internal metal isotope normalization beads and acquired on a Helios mass cytometer (Fluidigm Corporation) at a rate of 250–300 events per second. Purified antibodies were obtained from commercial vendors and labeled with metal isotopes using the MAXPAR™ X8 chelating polymer kit (Fluidigm Corporation) following the manufacturer’s instructions (Table 1). Acquired data was signal normalized and concatenated using the Helios Software version 7.0 (Fluidigm Corporation) and uploaded into Cytobank (Cytobank Inc., South San Francisco, CA, USA) for further QC data processing. Gaussian parameters of the Helios system were used for doublet exclusion, and Ir+ and Rh− signals were used to gate out dead cells and normalization beads. Data was transformed using an arcsinh (X/5) transformation. Single live intact cells were then used for data analysis.

**Flow cytometry analysis for M1 and M2 markers in monocytes**: Monocytes were seeded at 2 × 10^6^ cells/mL in 1 mL per well in 12-well plates, given that only a fraction of the isolated monocytes would adhere for subsequent experiments. After 24 h, 20 µL (~2 × 10^9^ particles) of N-F and MF-F sEVs was added and incubated for 24 h. The supernatant medium was removed and the attached cells were washed 3 times with PBS. Cells were dissociated with cell dissociation solution (SARTORIUS 223200712, Göttingen, Germany) for 15 min and washed with PBS. The cells were suspended in 100 uL of staining buffer and incubated for 1 h with the following conjugated antibodies: BV510 anti-CD14 (BD, 563079), R718 anti-CD16 (BD, 566969); PE-Cy7 anti-CD80 (BD, 561135), PE-CF594 anti-CD86 (BD, 562390); BV421 anti-CD163 (BD, 562643), APC anti-CD206 (BD, 550889), and BB515 anti-CD274 (BD n, 564554). Then the cells were washed and analyzed using a Galios Flow Cytometer.

**RNA extraction from sEVs and monocytes**: RNA was isolated from monocytes after incubation for 24 h with N-F or MF-F sEVs using Trizol (Life technologies, Carlsbad, CA, USA) and from N-F and MF-F sEVs using the Total sEV RNA isolation kit (Invitrogen by Life Technologies). The purity and concentration of purified RNA were determined using a Nanodrop.

**Real Time qPCR** (all reagents from Applied Biosystems, Foster City, CA, USA): Reverse transcription reaction: 5 ng of total RNA was used for reverse cDNA reaction using the TaqMan MicroRNA Reverse Transcription Kit. A total of 10 µL of RT reaction master mix was prepared on ice for each reaction, as follows: 0.8 µL of 100 mM dNTPs, 3.2 µL nuclease-free water, 2 µL 10× reverse transcription buffer, 1 µL RNase inhibitor (20 U/µL), 1 µL RT enzyme (50 U/µL) and 2 µL of RT random primers. Each sample was mixed gently, centrifuged at 4 °C and kept in ice. Then, 10 µL of each sample was loaded into well of a 96-well reaction plate. A total of 5 µL of total RNA (1 ng) was added to each well. Every well was pipetted carefully, and the plate was centrifuged briefly at 4 °C. The plate was then run in the StepONE plus Real-time PCR ABI-7000 Sequence Detection System and programmed for one cycle as follows: 10 min at 25 °C, 120 min at 37 °C, and 5 min at 85 °C. qRT-PCR: 2 µL cDNA was used as a template in a 10 µL PCR reaction. A PCR reaction for each sample was performed in triplicate, including blank control without cDNA. Every tube contained: 2.5 µL Nuclease-free water, 5 μL TaqMan PCR Master Mix and 0.5 µL specific Primer TM. Then, 8.0 µL from each reaction was loaded into wells of a 96-well reaction plate and 2 µL specific cDNA was added (2 µL Nuclease-free water was added to the control tube). The plate was centrifuged briefly at 4 °C and loaded into the Step One plus qRT-PCR system, programmed as follows: 10 min at 95 °C, 40 cycles of 20 s at 95 °C and 20 s at 60 °C. Primers for microRNAs: hsa-miR-23a-p primer (ID 002270), hsa-miR-27a-3p (ID 000408), hsa-miR-92 (ID 002269), and hsa-miR-1468-5p (ID 002271). miR expression in cell lines and cell line-derived sEVs were normalized to U6-snRNA (ID 001973, ABI). Primers for cytokines: IL-12α (HS 01073447), TGF-β (HS 00998133), IL-6 (HS 00985639), IL-1β (HS 00174097), and IL-10 (HS 00961622) normalized to HPRT (HS 99999909). Data analysis: The differential expression of specific miRNA was demonstrated in −ΔCt and RQ (relative quantification) for the fold change in the expression level of specific miRNAs in the study samples compared to the reference group as follows: –ΔCt = CtU6/HPRT-CtmiR/cytokines, and RQ = 2^−ΔΔCt^ = mean 2(−ΔCt tested group)/mean 2(−ΔCt reference group) presented with standard error (SE). All calculations were carried out with DataAssistTM, version 3.01 (Thermo Fisher). A non-parametric unpaired Mann–Whitney test was used for all the comparisons.

**Viability of T cells co-cultured with polarized monocytes**: Monocytes were seeded at 2 × 10^6^ cells/mL in 0.5 mL per well in 24-well plates, given that only a fraction of the isolated monocytes would adhere for subsequent experiments. After 24 h, 10 µL (~1 × 10^9^ particles) of N-F- or MF-F-derived sEVs was added per well for 24 h. Then the monocytes were washed and 500 µL of autologous CD4^+^ or CD8^+^ T cells (cultured in RPMI supplemented with IL-2) was added at a 1:5 monocytes: T cell ratio. T cell viability was assessed by trypan blue exclusion at 48 h and 72 h.

**Statistical analysis**: All experiments were performed at least 3 times, and data were analyzed with GraphPad Prism 9 (San Diego, CA, USA). The significance of the differential effects among the comparative groups were determined by using the two-tailed Student’s *t*-test/two-way ANOVA test. The variance was similar between compared groups. The signs for the *p*-values are: * for 0.01 < *p* < 0.05; ** for 0.005 < *p* < 0.01, *** for *p* < 0.005 and # for non-significance *p* > 0.05.

## 3. Results

### 3.1. Exosome-Enriched sEVs from MF Fibroblasts Reduced the Viability of Normal PBMCs

**Figure 1:**
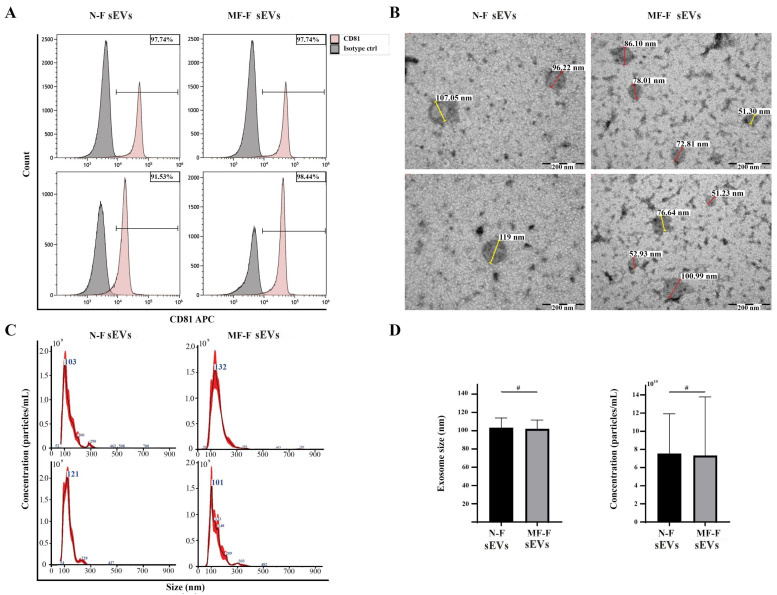
Characterization of sEVs derived from N-Fs and MF-Fs. (**A**) FACS analysis of CD81 compared to IGg1 APC isotype control on sEVs that were pre-conjugated to latex beads. (**B**) Representative TEM analysis of N-F- and MF-F sEVs (bar size: 200 nm). (**C**) Size distribution and vesicle concentration determined by NTA (samples diluted 1:1000). (**D**) Size average and concentration average based on NTA of sEVs of N-Fs (n = 8) and MF-Fs (n = 8). # for non-significance *p* > 0.05.

Fibroblasts from both MF skin biopsies (MF-Fs) and normal fibroblasts (N-Fs) from healthy donor skin were cultured, and their sEVs were isolated using differential centrifugation, filtration, and ultracentrifugation. Exosome-enriched sEV identity was confirmed by detecting CD81, a canonical exosomal surface marker, using flow cytometry analysis of exosome-coated latex beads (Figure 1A). The absence of calnexin, a negative exosomal marker, was verified by Western blot and compared with its expression in parental fibroblast cells (Supplementary Figure S1). Flow cytometry showed that 91–98% of the sEVs from both MF-Fs and N-Fs were CD81-positive, validating successful isolation of exosome-enriched sEVs. Transmission electron microscopy (TEM) confirmed membrane-enclosed EVs with diameters of 30–150 nm (Figure 1B). Nanoparticle tracking analysis (NTA) data of six samples of N-F sEVs and six samples of MF-F sEVs are shown in Supplementary Table S1, showing an average of ~10^11^ particles/mL. Representative NTA plots of particle concentration and size are shown (Figure 1C), along with their average mode diameters of 103.5 nm for N-F sEVs and 101.9 nm for MF-F sEVs, with a similar pick particle yield of 2 × 10^9^ particles/mL (Figure 1D). In accordance with the MISEV2023 recommendations, we refer to our EV preparations as sEVs rather than exosomes, as they are defined operationally by size (<200 nm by NTA/TEM) and isolation method (differential ultracentrifugation), rather than by demonstrated endosomal origin. The protein content of the isolated sEVs was quantified using a BCA kit, yielding a particle-to-protein ratio range of 1–2 × 10^8^ particles per µg of protein (Supplementary Table S1).

Mass spectrometry was employed for comprehensive proteomic analysis of sEVs derived from four MF-F and three N-F samples. High and comparable expression levels of CD81, CD63, CD9, TSG101, and PDCD6IP (positive exosome markers) were detected across all sEV samples, while Calnexin, Calreticulin, GRP, GP96, and GM130 (negative exosome markers) were absent. Nineteen proteins were significantly upregulated in MF-F compared to N-F sEVs (fold change ≥ 2), whereas only four proteins were significantly higher in N-F compared to MF-F sEVs (Supplementary Table S2).

### 3.2. MF-F and N-F sEVs Modulate Immune Cell Phenotype and Composition

**Figure 2:**
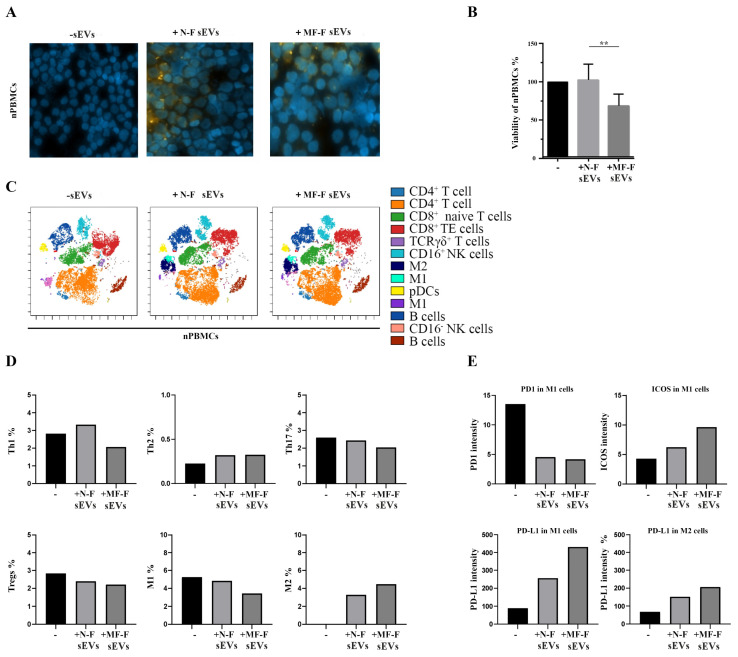
Internalization of N-F and MF-F sEVs along with their effects on nPBMC viability and exploratory immune cell modulation. (**A**) Fluorescence microscopy analysis for uptake of PKH26-labeled MF-F and N-F sEVs into nPBMCs after 24 h of incubation with the sEVs. (**B**) MTT viability assay for nPBMCs after 72 h incubation with different samples of MF-F (n = 7) and N-F (n = 7) sEVs. (**C**) Representative tSNE graph for immune cell subset of nPBMCs with and without N-F (n = 2) and MF-F (n = 2) sEVs for 24 h analyzed by cyTOF. Each cluster represents a different exploratory subpopulation of the immune cells. (**D**) Proportion of exploratory distinct immune cell subsets among CD45^+^ cells altered by N-F and MF-F sEVs in nPBMCs. (**E**) Expression of exploratory immune regulatory proteins in M1 and M2 monocytes, both with and without exposure to N-F and MF-F sEVs. ** for 0.005 < *p* < 0.01.

To qualitatively, rather than quantitatively, assess sEV internalization, nPBMCs from healthy donors (n = 3) were incubated for 24 h with PKH26-labeled sEVs from N-Fs and MF-Fs. The nPBMCs were then analyzed by fluorescence microscopy showing cytoplasmic focal PKH26 staining only in the cells with sEVs (Figure 2A and  Figure S2). The intensity of sEV uptake varied across different regions of the slide, with no consistent differences between MF-F- and N-F-derived sEVs, as exemplified in Figure 2A.

To evaluate the effect of sEVs on immune cell viability, nPBMCs were incubated with sEVs derived from N-Fs (n = 7) and MF-Fs (n = 7) for 72 h, and their viability was analyzed via MTT assay. nPBMCs exposed to MF-F sEVs showed significantly reduced viability compared with cells treated with N-F sEVs or untreated controls (*p* = 0.0045) (Figure 2B). This suggests that MF-F sEVs may contribute to immune cell depletion in the TME of MF.

To examine the effect of N-F and MF-F sEVs on immune cells, we performed single-cell flow mass cytometry on nPBMCs using a custom-made panel of 38 antibodies (Table 1). Fresh nPBMCs (n = 2) were incubated for 24 h with MF-F (n = 2) and N-F (n = 2) sEVs, then fixed, stained, and analyzed by mass cytometry. T-SNE analysis revealed distinct immune cell clusters following exposure to sEVs from different fibroblast sources (Figure 2C). Because this mass cytometry experiment included only two sEV samples per group, the results should be regarded as preliminary and exploratory, serving primarily as hypothesis-generating observations. These data suggest an apparent increase in Th2 cells after exposure to both N-F and MF-F sEVs, together with an apparent decrease in Th17 cells, M1 macrophages, Tregs, and PD-1 expression on M1 cells for sEVs from both fibroblast types, with a more pronounced effect for MF-F sEVs (Figure 2D). N-F sEVs tended to increase the proportion of Th1 cells, whereas MF-F sEVs tended to increase the proportion of M2 cells and to enhance PD-L1 expression on M1/M2 macrophages and ICOS expression on M1 cells (Figure 2E).

### 3.3. MF-F sEVs Promote M2 Cytokine Expression and PD-L1 Upregulation in Macrophages

**Figure 3:**
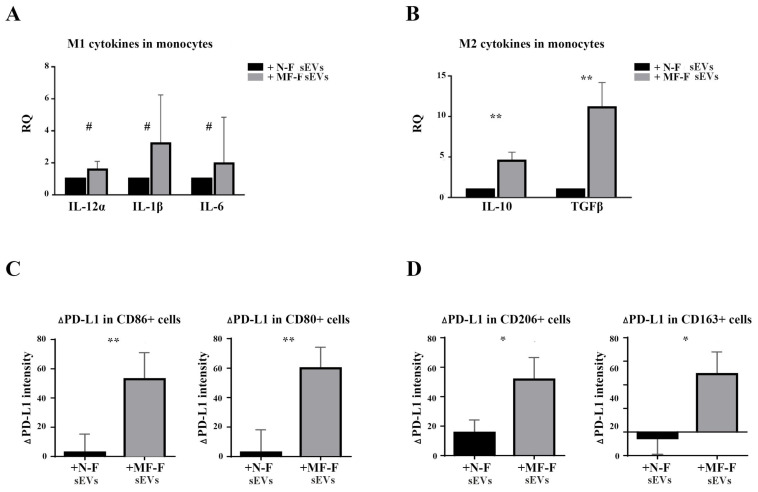
MF-F sEVs induce M2 polarization and PD-L1 upregulation in monocytes. (**A**,**B**) RNA was extracted from monocytes incubated with NF and (n = 5) MF-F (n = 5) sEVs for 24 h and analyzed by qRT-PCR for M1 and M2 cytokines. (**C**) The ∆ of PD-L1 expression (∆ = PD-L1 in cells with sEVs—PD-L1 in cells without sEVs) in CD86- and CD80-positive cells treated with NF sEVs (n = 5), and MF-F sEVs (n = 5). (**D**) The ∆ of PD-L1 expression (∆ = PD-L1 in cells with sEVs—PD-L1 in cells without sEVs) in CD163- and CD206-positive cells treated with NF sEVs (n = 5) and MF-F sEVs (n = 5). * for 0.01 < *p* < 0.05; ** for 0.005 < *p* < 0.01, and # for non-significance *p* > 0.05.

To further investigate the effect of MF-F sEVs specifically on monocytes, primary CD14^+^CD16^−^ monocytes were isolated from healthy blood donors (Supplementary Figure S3A) and were incubated for 24 h with MF-F (n = 5) and N-F (n = 5) sEVs. Flow cytometry analysis showed that both types of sEVs increased M1 markers (CD86^+^, CD80^+^) (Supplementary Figure S3B), which are associated with pro-inflammatory, anti-tumor effects [16], as well as M2 markers (CD163^+^, CD206^+^) (Supplementary Figure S3C), which are linked to anti-inflammatory, pro-tumor function [17] with a more pronounced effect observed for MF-F sEVs. qRT-PCR analysis of monocyte RNA confirmed that MF-F sEVs, compared with N-F sEVs, significantly upregulated M2-associated cytokines, IL-10 (*p* = 0.0079) and TGF-β (*p* = 0.0079) (Figure 3B), whereas the M1-associated cytokines IL-12α, IL-1β, and IL-6 showed no significant changes (*p* > 0.19) (Figure 3A).

PD-L1 expression was also analyzed by flow cytometry in the primary monocytes polarized by sEVs. PD-L1 levels were significantly higher in monocytes treated with MF-F-derived sEVs than in those treated with N-F-derived sEVs (Supplementary Figure S4). This was observed both within CD80^+^ and CD86^+^ cells (M1 phenotype; *p* = 0.0167 and *p* = 0.0091, respectively; Figure 3C) and within CD206^+^ and CD163^+^ cells (M2 phenotype; *p* = 0.0219 and *p* = 0.0145, respectively; Figure 3D). The proteomic analysis of sEV cargo derived from both N-F (n = 3) and MF-F (n = 4) cells, presented in Supplementary Table S2, did not detect PD-L1 proteins in any of the sEV samples. Several microRNAs, both extracellular and cellular, have been reported to associate with PD-L1 upregulation and M2 polarization, including miR-27a-3p [18], miR-23a-3p [19], miR-92 [20], and miR-1468-5p [21]. All four microRNAs were tested by qRT-PCR in sEVs derived from MF-Fs (n = 5) and N-Fs (n = 5), and no significant differences in expression were observed. 

### 3.4. Monocytes Polarized by MF-F sEVs Versus N-F sEVs Reduce CD4^+^ and CD8^+^ T Cell Viability

**Figure 4:**
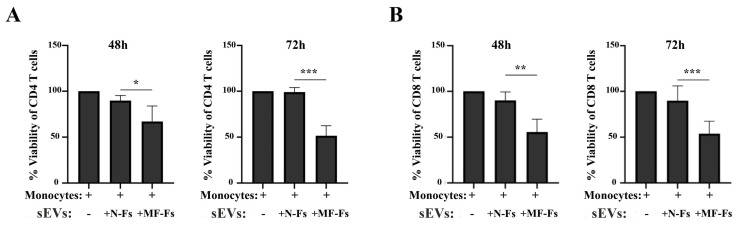
MF-F sEVs reduced the viability of CD4^+^ T helper cells and CD8^+^ cytotoxic T cells. (**A**) Trypan blue viability assay for CD4^+^ T helper cells following co-culture with monocytes pre-polarized by MF-F sEVs (n = 4) vs. N-F sEVs (n = 4) for 48 h and 72 h. (**B**) Trypan blue viability assay for CD8^+^ cytotoxic T cells following co-culture with monocytes pre-polarized by MF-F sEVs vs. N-F sEVs for 48 h and 72 h. * 0.01 < *p* < 0.05; ** 0.005 < *p* < 0.01, *** *p* < 0.005.

M2 monocytes and PD-L1 are known to suppress T cell proliferation and activity [22,23,24,25]. Therefore, we analyzed the effect of primary monocytes polarized by MF-F sEVs vs. N-F sEVs on the viability of T helper cells and on cytotoxic T cells. CD4^+^ T cells, CD8^+^ T cells, and primary monocytes were isolated from autologous blood samples of healthy donors, yielding 86.6% CD4^+^ T cells and 95.5% CD8^+^ T cells (Supplementary Figure S5A,B respectively). The monocytes were incubated for 24 h with MF-F sEVs (n = 4) and N-F sEVs (n = 4), and then co cultured with autologous T cells for 48 and 72 h. T cell viability assessed by Trypan blue exclusion assay, which showed that CD4^+^ T cell viability was significantly reduced when co-cultured with monocytes polarized by MF-F sEVs compared with monocytes polarized by N-F sEVs (approximately 30% reduction at 48 h, *p* = 0.0422; and 50% reduction at 72 h, *p* = 0.0002) (Figure 4A). CD8^+^ T cells co-cultured with monocytes polarized by MF-F sEVs, compared to those with monocytes polarized by N-F sEVs, exhibited a ~40% decrease in viability at both 48 and 72 h (*p* = 0.0063, *p* = 0.0148, respectively) (Figure 4B). These findings suggest that MF-F sEVs promote an immunosuppressive environment by driving monocyte polarization towards M2 macrophages with high PD-L1 expression, thereby impairing T cell viability.

## 4. Discussion

While the immunosuppressive effects of fibroblast-derived sEVs, particularly exosomes, have been extensively studied in several types of cancer [20], little is known about the role of MF fibroblast-derived sEVs in promoting immune suppression in CTCL. Here, we propose that N-F exosome-enriched sEVs favor an anti-tumor response, whereas MF-F exosome-enriched sEVs promote immune suppression by increasing pro-tumoral M2 macrophages with high PD-L1 expression and by tending to upregulate Th2, Th17, and Treg cells while downregulating anti-tumoral Th1 cells. The immunosuppressive role of MF-F sEVs is consistent with our previous study showing the CAFs in MF are predominantly FAP^+^CXCL12^+^SMA [9], a phenotype now recognized as immunosuppressive CAFs (iCAFs) [26], as further supported by the present data.

A common feature of cancer progression is the transition from M1 to M2 macrophages, characterized by a decrease in M1 macrophages and an increase in M2 macrophages [27]. The present study showed that MF-F sEVs reduced M1-like macrophages and increased M2 macrophages. PD-L1 expression was also increased on both M1 and M2 macrophages following exposure to MF-F sEVs. M1 macrophages produce pro-inflammatory cytokines (IL-12, TNF-α) with an anti-cancer effect, whereas M2 macrophages secrete anti-inflammatory cytokines (IL-10, TGF-β) [28] with a pro-tumoral effect. In many tumors, M2 macrophages predominate as the TME is enriched with factors like IL-4, IL-13, TGF-β, and IL-10 that promote M2 polarization, dampen immune responses and support tumor growth [29]. Querfeld et al. demonstrated that M2 macrophages increased when monocytes were exposed to CTCL cell supernatants [30], which contain both cytokines and sEVs. Additionally, Sugaya et al. reported higher numbers of CD163^+^ (M2) macrophages in CTCL lesions compared with healthy skin, identifying CD163 as a dominant marker for tumor-associated macrophages (TAMs) in MF [31]. Our findings showed that although both N-F and MF-F sEVs upregulated M1 and M2 macrophage markers, MF-F sEVs significantly increased M2-associated cytokines (IL-10, TGF-β) without altering M1-associated cytokines (IL-12α, IL-1β, IL-6). This suggests that whereas N-F sEVs may physiologically support both M1 and M2 polarization, MF-F sEVs drive a more pronounced shift towards an immunosuppressive M2 phenotype. These observations are in line with previous studies linking macrophage polarization to cancer progression and indicate that tumor-derived sEVs contribute to this shift. Targeting the M1/M2 balance may therefore represent a promising therapeutic strategy, as recent work has explored macrophage-polarization agents such as IFN-γ, CSF1R inhibitors and TLR agonists to improve cancer treatment [32].

Moreover, we showed that M2-polarized monocytes with high PD-L1 expression, induced by MF-F sEVs rather than by N-F sEVs, directly suppress the viability of T helper and cytotoxic T cells. Our findings are consistent with previous reports demonstrating that M2 macrophages suppress both CD4^+^ (T helper) and CD8^+^ (cytotoxic) T cell proliferation through the interaction of PD-L2 on M2 macrophages and PD-1 on T cells [33]; that high PD-L1 expression on M2 macrophages engages with PD-1 on T cells, leading to exhaustion and apoptosis of both cytotoxic and helper T cells [34]; and that M2 macrophages secrete IL-10 and TGF-β to create immunosuppressive conditions that further impair T cell viability [34].

Several studies on CAF-derived exosomes/sEVs support our observations on M2 macrophage polarization and increased PD-L1 expression. CAF exosomes/sEVs promote M2 macrophage polarization by activating the PTEN/PI3Kγ signaling pathway, resulting in pro-tumoral macrophage function and an immunosuppressive microenvironment in pancreatic cancer [35]; CAF exosomes/sEVs that carry specific long noncoding RNAs induce an M2 macrophage phenotype and tumor-supportive immune profiles in hepatocellular carcinoma [36]; CAF exosomes/sEVs carrying OIP5-AS1/miR-142-5p increase PD-L1 expression in lung cancer cells, thereby facilitating immune suppression [37]; and CAF exosomes/sEVs enriched in miR-146a promote M2 macrophage polarization in bladder cancer, enhancing an immunosuppressive microenvironment that favors tumor progression [38]. In addition, CAF-derived exosomes/sEVs enriched with miR-92 directly induce M2-type macrophage markers and are associated with increased PD-L1 expression in both macrophages and breast cancer cells, thereby supporting immune evasion [21].

We also did not detect PD-L1 proteins in our MF-F sEVs, even though these sEVs induced PD-L1 expression in recipient monocytes, presumably by delivering microRNAs that indirectly upregulate PD-L1.

Because increased Th2 and suppressed Th1 cytokines are known to correlate with MF progression [39,40], we examined the effect of MF-F sEVs on these T cell subsets. The effects of MF-F sEVs versus N-F sEVs and the differences between them were modest in the mass flow cytometry assay, likely due to the exploratory nature of the experiment and the use of nPBMCs rather than purified T cell subsets. Overall, MF-F sEVs compared with N-F sEVs tended to reduce the proportion of Th1 (anti-tumoral) cells within nPBMCs and slightly increase the proportion of Th2 (pro-tumoral) cells.

Th17, a CD4^+^ T cell subset that produces IL-17, is crucial for defense against fungal and bacterial but also contributes to autoimmune diseases [41]. The role of Th17 cells, and IL-17 in cancer is context dependent; IL-17 can recruit immune cells to tumor sites and enhance CD8^+^ T-cell activation, yet in CTCL, Th17 cells may either support or inhibit tumor growth [42]. Some studies suggest that benign T cells in CTCL lesions lose Th17 characteristics, reducing their anti-tumor potential [43]. We observed a modest decreasing trend in Th17 cells after exposure to N-F and MF-F sEVs, with a slightly stronger reduction trend induced by MF-F sEVs.

Tregs promote immune tolerance for inhibiting anti-tumor immunity and limiting tumor-promoting inflammation [44]. In MF, the Tregs are present within the malignant skin infiltrates, where they promote immune evasion and disease progression and correlate with advanced MF stages [45]. In our system, however, we observed a slight decreasing trend in Tregs after exposure to both MF-F and N-F sEVs. This pattern suggests that the observed effect may reflect a normal regulatory function response rather than a malignant influence, potentially related to heterogeneity within the primary fibroblast cultures.

This study has several limitations. First, our isolated exosome/sEV preparations may contain other small EV subtypes rather than pure exosomes and should therefore be considered an exosome-enriched sEV fraction. Second, the immune-subset changes detected by CyTOF are based on a small number of donors and should be regarded as exploratory. Third, the impact of MF-F sEV-polarized monocytes on T cells was assessed only at the level of cell viability and was not comprehensively assessed in terms of T cell proliferation, activation, or effector function.

Advanced studies in MF patient samples in vivo are required to confirm our findings and may ultimately allow identification of patients with a more immunosuppressive TME and thus potentially poorer prognosis or higher risk of progression, based on a high MF-F sEV load or a strongly M2-polarizing sEV signature. Localized strategies targeting MF-CAF-derived sEVs by inhibiting their release or uptake, or by modulating sEV PD-L1 and/or miRNA cargo, could help reverse M2 polarization and restore T cell function. In parallel, such in vivo studies could guide the development of therapeutic approaches aimed at counteracting these effects, for example by blocking sEV release or uptake, altering sEV cargo, or reprogramming M2 macrophages to re-establish anti-tumor immunity.

## 5. Conclusions

This is the first study to investigate sEVs derived from primary fibroblasts of MF lesions. MF fibroblast-derived sEVs promote a tumor-supportive microenvironment by inducing M2-like macrophage polarization and upregulating PD-L1 expression, both of which reduce the viability of CD4^+^ helper T cells and CD8^+^ cytotoxic T cells. These findings not only advance our understanding of the molecular mechanisms shaping the MF TME but also highlight the therapeutic potential of targeting sEV pathways and PD-L1 in the treatment of MF.

## Figures and Tables

**Table 1:** Antibodies for cyTOF analysis.

Target	Metal	Catalog	Clone	Vendor
Anti-Human CD4	174Yb	3174004B	SK3	Fluidigm
Anti-Human CD8	168Er	3168002B	SK1	Fluidigm
Anti-Human CD197/CCR7	167Er	3167009A	G043H7	Fluidigm
Anti-Human CD45RO	165Ho	3165011B	UCHL1	Fluidigm
Anti-Human CD45RA	155Gd	3155011B	HI100	Fluidigm
Anti-Human CD45	89Y	3089003B	HI30	Fluidigm
Anti-Human CD183 [CXCR3]	163Dy	3163004B	G025H7	Fluidigm
Anti-Human CD196/CCR6	141Pr	3141003A	G034E3	Fluidigm
Anti-Human CD197/CCR7	167Er	3167009A	G043H7	Fluidigm
Anti-Human CD25	169Tm	3169003B	2A3	Fluidigm
Anti-Human CD127/IL-7Ra	143Nd	3143012B	A019D5	Fluidigm
Anti-Human CD19	142Nd	3142001B	HIB19	Fluidigm
Anti-Human CD3	154Sm	3154003B	UCHT1	Fluidigm
Anti-Human CD56	176Yb	3176009B	N901	Fluidigm
Anti-Human CD161	164Dy	3164009B	HP-3G10	Fluidigm
Anti-Human CD123/IL-3R	151Eu	3151001B	6H6	Fluidigm
Anti-Human CD16	148Nd	3148004B	3G8	Fluidigm
Anti-Human HLA-DR	173Yb	3173005B	L243	Fluidigm
Anti-Human CD14	175Lu	3175015B	M5E2	Fluidigm
Anti-Human CD86/B7.2	150Nd	3150020B	IT2.2	Fluidigm
Anti-Human CD163	145ND	3145010B	GHI/61	Fluidigm
Anti-Human TCRgd	152Sm	3152008B	11F2	Fluidigm
Anti-Human CD185/CXCR5	153Eu	3153020B	RF8B2	Fluidigm
Anti-Human CD66b	162Dy	3162023B	80H3	Fluidigm
Anti-Human CD20	171Yb	3171012B	2H7	Fluidigm
Anti-Human CD38	144Nd	3144014B	HIT2	Fluidigm
Anti-Human CD11c	147Sm	3147008B	Bu15	Fluidigm
Anti-Human PD-L1	156Gd	3156026B	29E.2A3	Fluidigm
Anti-Human CD279/PD1	172 Yb	201811-100	NAT105	Abcam
Anti-Human CTLA4	170Er	3170005B	14D3	Fluidigm
Anti-Human CXCR4	Ce 140	271934-100	EPUMBR3	Abcam
Anti-Human CD366 Tim 3	159Tb	3159037B	F38-2E2	Fluidigm
Anti-Human CD134 OX40	158Gd	3158012B	ACT35	Fluidigm
Anti-Human CD278/ICOS	157 Gd	237562-100	EPR22181	Abcam
Anti-Human CD335-NKp46	166 Er	BLG-331902	9E2	BLG
C Anti-Human D30	146 Nd	213047-100	CD30/412	Abcam
Anti-Human CD223/LAG-3	ln 115	227579-100	EPR20261	Abcam
Anti-Human CD47	209Bi	3209004B	CC2C6	Fluidigm

## Data Availability

The data that support the findings of this study are available from the corresponding author, [L.M.], upon reasonable request.

## Supplementary Materials

The following supporting information can be downloaded at: https://www.mdpi.com/article/10.3390/cancers18132140/s1, Figure S1: Absence of calnexin, a negative exosomal marker, in MF-F and N-F sEVs; Figure S2: MF-F and N-F sEV internalization to nPBMCs; Figure S3: MF-F sEVs polarize monocytes towards M1 and M2 cells; Figure S4: MF-F sEVs upregulate the expression of PD-L1 in monocytes; Figure S5: Verification of CD4^+^ and CD8^+^ T cell isolation from blood of healthy donor; Table S1. (A): MF-F sEV size distribution and concentration based on NTA and BCA protein quantification; (B): N-F sEV size distribution and concentration based on NTA and BCA protein quantification. Table S2: Differential protein expression in MF-F vs. N-F sEVs based on proteomic mass spectrometry.
