# Supplementary material for: Fibroblast-Derived Small Extracellular Vesicles Promote M2 Macrophage Polarization and PD-L1 Upregulation in Mycosis Fungoides
Source: Cancers (Basel). 2026 Jul 2;18(13):2140. doi: 10.3390/cancers18132140 (PMC13360305; doi:10.3390/cancers18132140)
Supplement: Supplementary file 1 [file cancers-18-02140-s001.zip › cancers-4312905-supplementary.pdf]

Sup Table S1A: MF-F sEV size distribution and concentration based on NTA and BCA protein quantification

sEV sample	Mean ± SD (nm)	Mode (nm)	D10 (nm)	D50 (nm)	D90 (nm)	Concentration (particles/mL)	Particles/µg protein
MF-F 23	137.8 ± 5.8	100.5	89.1	123.6	204.1	9.90×10^{10}	1.50×10^{8}
MF-F 24	131.1 ± 13.4	126.3	111.5	152.1	243.6	1.10×10^{11}	1.70×10^{8}
MF-F 25	123.9 ± 2.9	96.5	80.4	108.3	170.6	8.50×10^{10}	1.10×10^{8}
MF-F 26	150.9 ± 6.4	95.5	94.3	139.9	212.8	8.70×10^{10}	1.10×10^{8}
MF-F 27	119.2 ± 3.0	86.4	82.5	110.6	174.6	8.60×10^{10}	1.80×10^{8}
MF-F 28	115.0 ± 18.9	94.1	49.8	107.5	184.8	8.90×10^{10}	2.00×10^{8}

Sup Table S1B: N-F sEV size distribution and concentration based on NTA and BCA protein quantification

sEV sample	Mean ± SD (nm)	Mode (nm)	D10 (nm)	D50 (nm)	D90 (nm)	Concentration (particles/mL)	Particles/µg protein
N-F 3	138.2 ± 13.5	101.8	74.6	118.1	212.9	8.50×10^{10}	2.00×10^{8}
N-F 11	132.1 ± 1.7	102.6	89.0	117.9	187.2	8.90×10^{10}	1.90×10^{8}
N-F 13	126.4 ± 2.5	120.9	91.2	121.0	168.0	1.30×10^{11}	1.30×10^{8}
N-F 14	137.5 ± 6.3	105.5	86.3	121.5	208.0	1.10×10^{11}	1.50×10^{8}
N-F 16	139.6 ± 3.5	111.0	88.5	128.9	206.3	8.80×10^{10}	1.50×10^{8}
N-F 19	138.3 ± 4.4	118.0	90.9	125.5	180.7	1.10×10^{11}	1.70×10^{8}

Sup Figure S1: Absence of calnexin, a negative exosomal marker, in MF-F and N-F sEVs

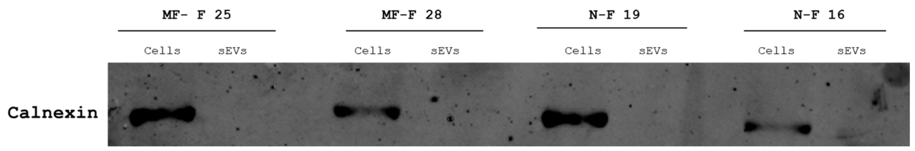

20 µg of total protein lysate from fibroblast cells and their matched sEVs were separated by SDS–PAGE, and the membrane was probed with an anti-calnexin antibody.

Sup Table S2: Differential protein expression in MF-F vs N-F sEVs based on proteomic mass spectrometry

Sup Table S2A: Proteins significantly higher in MF-F (n=4) versus N-F sEVs (n=3) (Fold change ≥ 2, p<0.05)

Gene name	p-value	Fold change
TF	0.016	2.110
COL6A1	0.002	2.230
PTGFRN	0.031	2.321
RAB2A;RAB2B	0.008	2.384
KEL	0.037	2.470
CD81	0.040	2.965
PSMB4	0.040	3.921
GPX3	0.011	3.997
C8B	0.038	4.031
HLA-C	0.041	4.044
PEX11G	0.022	4.105
PTX3	0.036	4.512
LRRC32	0.001	4.937
ANGPTL2	0.038	4.958
RBP4	0.041	5.395
FAT1	0.019	5.631
NLRP1	0.012	5.880
GAS8	0.033	6.357
KRT23	0.000	7.334

Sup Table S2B: Proteins significantly higher in N-F (n=3) versus MF-F sEVs (n=4) (Fold change ≥ 2, p<0.05)

Gene name	p-value	Fold change
SFN	0.040069	2.013
DARS	0.046203	4.389
TAGLN	0.000327	5.448
HSPA5	0.004394	6.156

Sup Figure S2: MF-F and N-F sEV internalization to nPBMCs

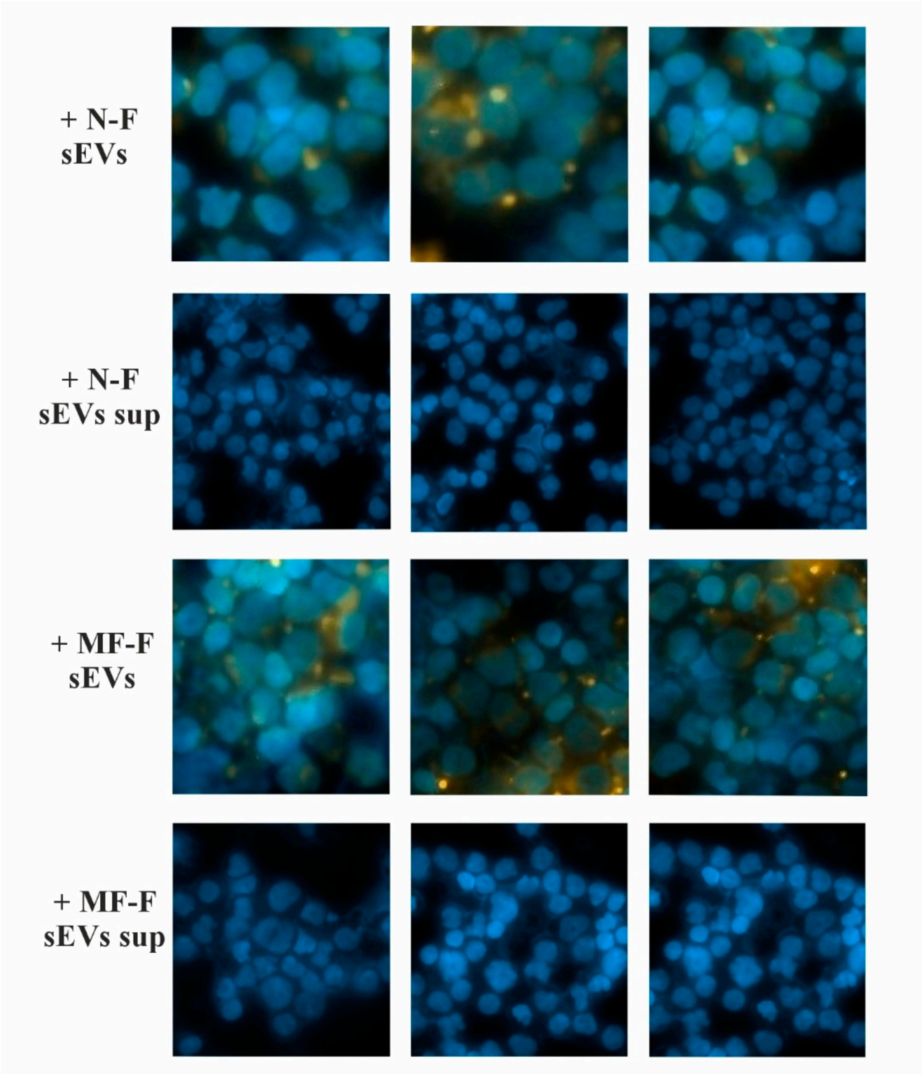

Fluorescence microscopy analysis for uptake of PKH26-labeled MF-F and N-F sEVs into nPBMCs cells after 24 hours of incubation with the sEVs and sEV wash supernatant.

Sup Figure S3: MF-F sEVs polarize monocytes towards M1 and M2 cells

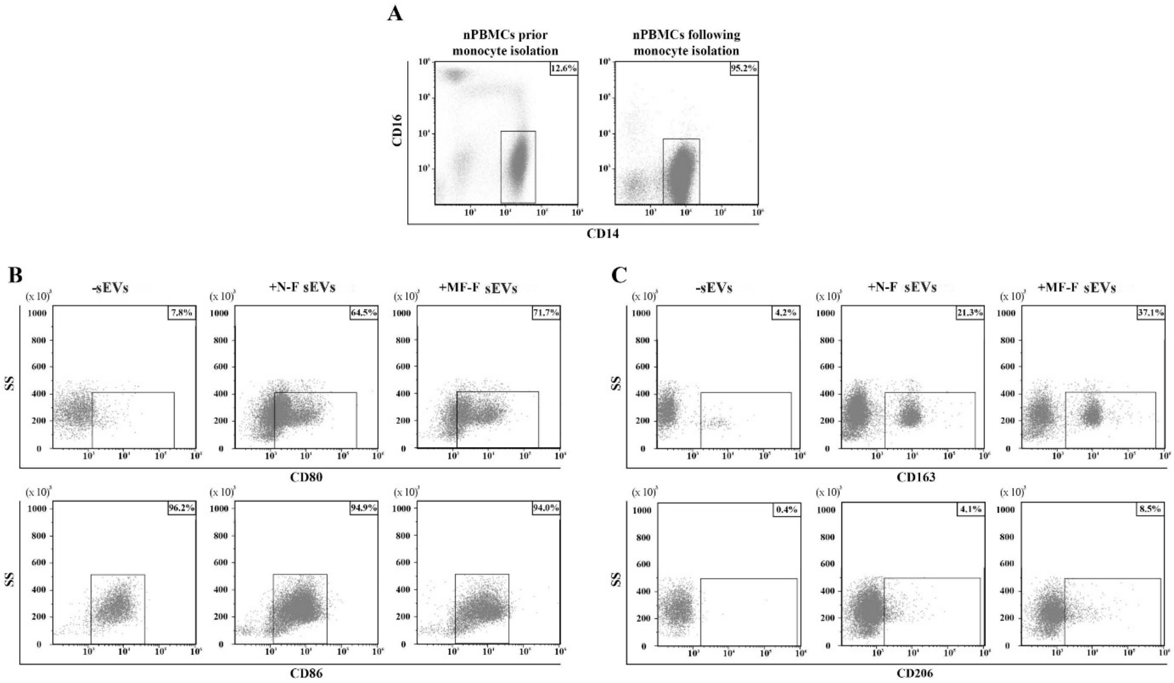

(A) Monocytes were isolated from nPBMCs using the EasySep magnetic beads kit and analyzed by FACS for CD14 and CD16 (BV510, R718) expression before and after isolation. **(B)** FACS plot for the expression of M1 marker (CD80 and CD86) in monocytes with or without N-F and MF-F sEVs for 24hr. **(C)** FACS plot for the expression of M2 markers (CD163 and CD206) in monocytes with or without N-F and MF-F sEVs for 24hr.

Sup Figure S4: MF-F sEVs upregulate the expression of PD-L1 in monocytes

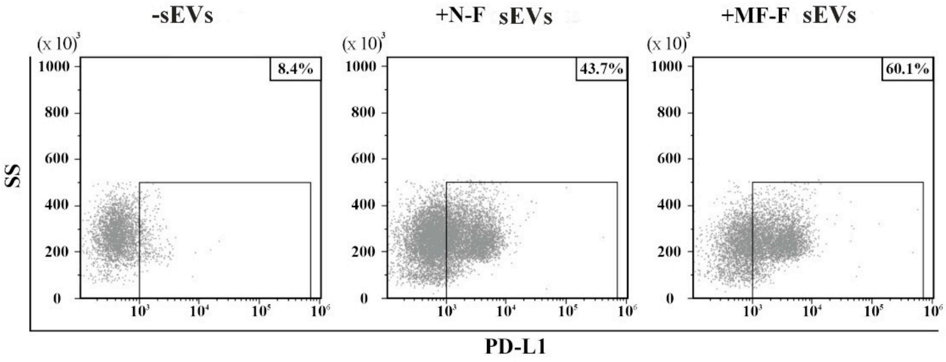

Representative FACS plot of PD-L1 in expression in monocytes that were pre incubated with and without N-F and MF-F sEVs for 24hr.

Sup Figure S5: Verification of $CD4^+$ and $CD8^+$ T cell isolation from blood of healthy donor

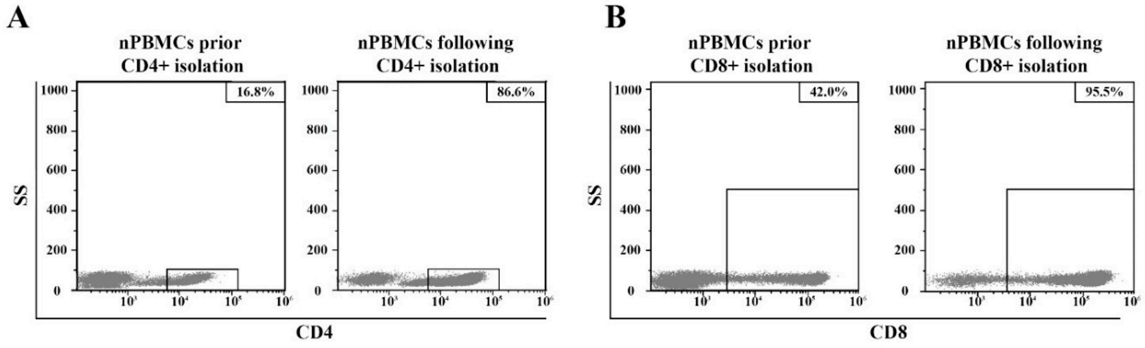

(A) FACS analysis showing the percentage of CD4+ (FITC) cells in nPBMCs: before and after CD4+ isolation using Rosettesep human CD4+ T cell enrichment cocktail. **(B)** FACS analysis showing the percentage of CD8+ (APC-VIO 770) cells in nPBMCs: before and after CD8+ isolation using Rosettesep human CD8+ T cell enrichment cocktail.
